# 肺类癌的病理特质与临床决策

**DOI:** 10.3779/j.issn.1009-3419.2013.05.06

**Published:** 2013-05-20

**Authors:** 蕾 朱, 屠阳 申, 杰 张, 小红 范

**Affiliations:** 1 200030 上海，上海交通大学附属胸科医院病理科 Department of Pathology, Shanghai Chest Hospital, Shanghai Jiao Tong University, Shanghai 200030, China; 2 200030 上海，上海交通大学附属胸科医院胸外科 Department of Thoracic Surgery, Shanghai Chest Hospital, Shanghai Jiao Tong University, Shanghai 200030, China; 3 200030 上海，上海交通大学附属胸科医院肺内科 Department of Pulmonary, Shanghai Chest Hospital, Shanghai Jiao Tong University, Shanghai 200030, China

**Keywords:** 肺肿瘤, 类癌, 病理特质, 临床处理, Lung neoplasms, Carcinoid, Pathological characteristic, Clinical management

## Abstract

**背景与目的:**

肺类癌采取以手术为主的多学科治疗，准确及时的病理诊断至关重要。本研究探讨肺类癌的病理特质，结合回顾性分析患者的转归预后，为临床决策提供依据。

**方法:**

收集支气管肺内发生的类癌手术标本32例，回顾性分析患者相关的临床病理资料，系统研究病灶病理学表现与临床诊断和治疗效果的相关性。

**结果:**

32例肺类癌患者中，典型类癌18例，不典型类癌14例；男女性别比为2.2:1；平均年龄（44±15）岁；近半数患者无症状；肿瘤最大径（3.1±1.3）cm；绝大多数为Ⅰ期患者（84.4%, 27/32），余Ⅱa期2例，Ⅲa期2例，Ⅳ期1例；随访时间为5.2年-9.7年；其中典型类癌随访15例，5年无进展生存率为100%；不典型类癌随访12例，5年无进展生存率为92.9%；肺类癌病理组织学的特质是典型的神经内分泌形态伴细胞角蛋白阳性，嗜铬素A、突触素和CD56等神经内分泌指标的表达，Ki-67指数的高低有助于鉴别诊断。

**结论:**

肺类癌的病理鉴别诊断应结合相关酶标染色，积极争取手术是适宜的临床决策。

## Abstract (en)

**Background and objective:**

Treatment of pulmonary carcinoid is a comprehensive modality focusing on surgery. Thus, accurate and timely preoperative and intraoperative pathological diagnoses are very crucial. The aim of this study is to retrospectively analyze the pathological characteristics of pulmonary carcinoid, its natural history, and patterns of disease progression to obtain evidence for clinical decision making.

**Methods:**

Clinical and pathological data of 32 patients with pulmonary carcinoid were retrieved and retrospectively analyzed. The pathological characteristics and their relationship with clinical diagnosis and treatment effect were systematically studied.

**Results:**

Among the 32 patients, 18 had typical carcinoid and 14 had atypical carcinoid. The male-to-female ratio was 2.2:1, and the average age was (44±15) years. Almost half of the patients were discovered by accident. The average maximum diameter was (3.1±1.3) cm. About 27 of all 32 cases (84.4%) were stage Ⅰ, 2 were stage Ⅱa, 2 were stage Ⅲa, and 1 was stage Ⅳ. The follow-up time was 5.2-9.7 years. The 5-year progression free survival of 15 typical carcinoid patients and 12 atypical carcinoid patients were 100% and 92.9%, respectively. The characteristic neuroendocrine morphology and variable expression levels of cytokeratin, chromogranin A, synaptophysin, CD56, and index of Ki-67 were important factors that differentiated pulmonary carcinoid from other tumors.

**Conclusion:**

Pathological diagnosis of pulmonary carcinoid must combine morphology with immunohistochemistry. Early-stage patients can also achieve good effect after surgery.

在外科切除的浸润性肺恶性肿瘤中，支气管肺类癌占1%-2%^[1]^。近30年，随着病理诊断、支气管内镜以及影像学技术的发展，支气管和肺的类癌明显增加^[2]^。该类肿瘤的影像学特征不典型，手术切除疗效满意。但活检和术中冰冻易于误诊，因而可能导致治疗决策的失当，因此准确的病理诊断至关重要。本研究旨在探讨肺类癌的组织病理学特质及其鉴别诊断要点，结合分析患者的自然病程、疾病进展模式和临床病理特征，期望为临床处理决策的制定提供病理学依据。

## 1 资料与方法

### 1.1 病例资料

2003年1月-2007年12月，共有32例肺类癌患者在上海市胸科医院就治，患者入院常规检查，要求达到：KPS≥80分；心电图无明显异常；肺功能正常；颅脑CT、腹部B超、骨ECT排除远处转移。所有患者均行肿瘤所在肺叶切除并行系统性淋巴结清扫。患者术后末次随访时间为2013年2月，随访时间 > 5年。患者住院资料以上海市胸科医院病案室存档病史资料为准。生存随访资料均来源于上海市疾病预防控制中心。通过SPSS 18.0数据库采集以下数据：住院号、性别、年龄、诊断时年龄、手术日期、手术类型、病理类型、T分期、N分期、清扫淋巴结分期组（数）、阳性淋巴结组，术后化疗方案及周期。

### 1.2 病理研究

本组手术标本共32例，重新复习所有患者的病理组织学切片。所有切片均由新鲜组织经10%中性福尔马林固定，常规取材、脱水、浸蜡、包埋、切片后进行HE染色。按照2004年WHO肺、胸膜、胸腺及心脏肿瘤病理学和遗传学分类^[3]^由两位高年资专科病理医生进行病理组织学分类，如诊断有分歧，则在多头显微镜下共同阅片以达到共识。需要行免疫酶标进行鉴别诊断的病例均使用罗氏全自动免疫组化机, 并经必要的抗原修复，一抗分别选取以下指标[细胞角蛋白（cytokeratin, CK）-AE1/3，1:200；波形蛋白（vimentin, VIM），1:300；突触素（synaptophysin, SYN），1:50；白细胞共同抗原（leukocyte common antigen, LCA），1:200；Ki-67，1:200；CK7，1:200；CK20，1:200；以上由上海长岛生物技术有限公司提供。嗜铬素A（chromogranin A, CGA），1:200；CD56，1:200；以上由DAKO公司提供。甲状腺转录因子1（thyroid transcription factor-1, TTF-1），1:200；Novocastra公司提供]。临床分期采用2009年国际抗癌联盟（International Union Against Cancer, UICC）和国际肺癌研究会（International Association for the Study of Lung Cancer, IASLC）公布的第7版肺癌国际分期^[4]^。

### 1.3 统计学方法

所有数据的统计分析均采用SPSS 18.0统计软件包进行处理。定量资料的分析采用*t*检验，分析两个总体率或构成比之间有无差异采用χ^2^检验。*P* < 0.05为差异具有统计学意义。

## 2 结果

### 2.1 一般临床特征

#### 2.1.1 性别差异

32例患者中，男性22例，女性10例，男女性别比为2.2:1。典型类癌18例，不典型类癌14例，均为男性高发。

#### 2.1.2 发病年龄

为9岁-76岁，平均年龄（44±15）岁，其中典型类癌平均年龄43岁，不典型类癌平均年龄45岁，两组无统计学差异（*P*=0.729）。

#### 2.1.3 临床症状

近1/2的类癌患者为体检时偶然发现的。最常见的症状包括咳嗽和咯血等，与支气管阻塞有关；肿瘤大小：跨度1 cm-6 cm，平均（3.1±1.3）cm，其中典型类癌平均直径2.9 cm，不典型类癌3.3 cm，两组无统计学差异（*P*=0.366）。

#### 2.1.4 好发部位

12例发生在左肺（左总支1例，左上肺5例，左下肺6例），20例发生在右肺（右总支3例，右上叶7例，右中叶2例，右中下叶1例，右下叶7例）。发生在段及段以上支气管者23例，其余分布于肺周围。

#### 2.1.5 病理分期

总体Ⅰ期患者占84.4%（27/32）。典型类癌Ⅰa期11例、Ⅰb期5例、Ⅱa期1例、Ⅲa期1例。不典型类癌Ⅰa期5例、Ⅰb期6例、Ⅱa期1例、Ⅲa期1例、Ⅳ期1例。两组无统计学差异（*P*=0.577）。

#### 2.1.6 随访时间

本组病例随访时间为5.2年-9.7年，其中典型类癌共随访15例，不典型类癌共随访12例。

#### 2.1.7 疗效预后

典型类癌患者全部存活，且无复发。其中2例患者进行了术后化疗，尚有1例患者因术前气管镜误诊小细胞癌，施行术前化疗。不典型类癌目前全部存活，其中4例患者术后化疗，2例患者因肿瘤距切端近并侵犯肺门血管、心包而行术后放疗且均无复发，1例Ⅳ期患者术后实施放、化疗和介入治疗，持续进展。

### 2.2 病理组织学特点

肺类癌肉眼标本多为支气管管壁浸润型或支气管腔内型肿块，少数为周围型病变，一般境界较清楚，或呈分叶状。典型和不典型类癌两者显微镜下可呈器官样（图 1A）、小梁状、岛状、栅栏状、带状或菊心团样排列，瘤细胞多呈多角形，极少情况下呈梭形；核染色质呈细颗粒状，少数略粗糙；典型类癌核仁一般不明显；胞浆呈少-中度嗜酸性细颗粒状，个别病例肿瘤细胞胞浆透亮；核浆比例小。少数病例细胞的异型性较明显。典型类癌无坏死，一般核分裂少于2个/2 mm^2^（10 HPF）。不典型类癌可伴坏死，一般核分裂（2-10）个/2 mm^2^（10 HPF）（图 1B）。免疫酶标提示CK绝大多数阳性，CGA、SYN、CD56可不同程度阳性（图 1C），Ki-67指数在典型类癌和不典型类癌中的表达各不相同。典型类癌Ki-67指数较低（图 1D），不典型类癌的Ki-67指数偏高（图 1E）。

**1:**
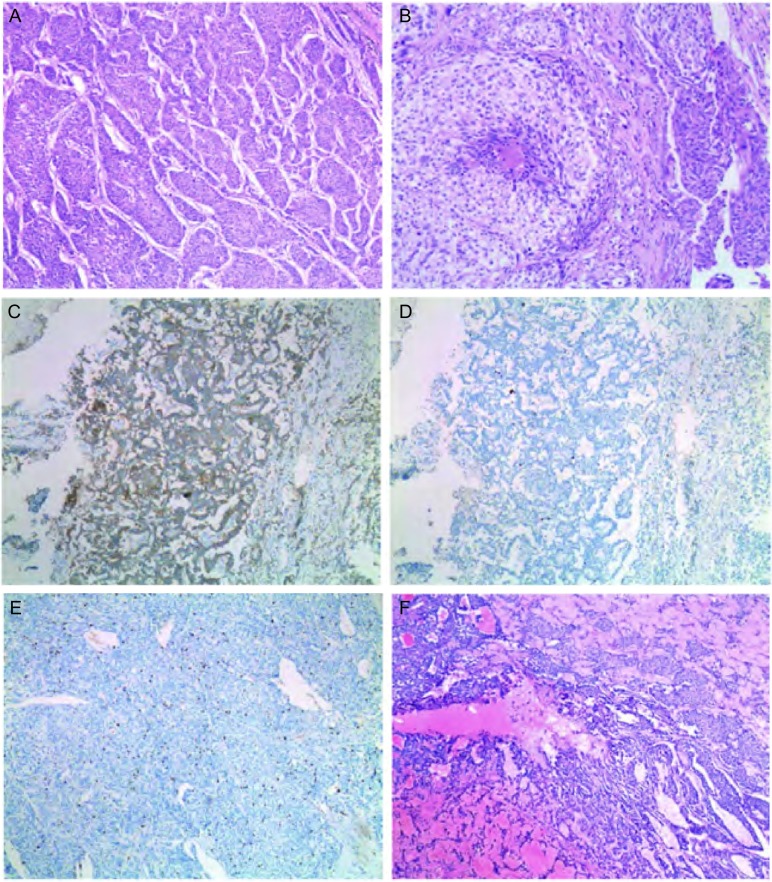
肺类癌的病理表现。A：肺典型和不典型类癌中最常见的器官样结构生长方式。肿瘤细胞较一致，胞浆中等量，嗜酸性，核呈细颗粒状（HE, ×40）；B：肺非典型类癌可出现灶状坏死（HE, ×40）；C：免疫组化显示类癌嗜铬素A阳性（×40）；D：本例典型类癌Ki-67指数约为2%（×40）；E：本例不典型类癌Ki-67指数约为10%（×40）；F：本例类癌组织学形态类似硬化性血管瘤（HE, ×40）。 The pathological pattern of lung carcinoid. A: An organoid growth pattern is the most common histologic pattern in both typical and atypical carcinoid. Tumor cells have uniform cytologic features with a moderate amount of eosinophilic cytoplasm. Nuclear features usually consist of finely granular chromatin; B: The necrosis usually consists of small punctate foci in atypical carcinoid; C: Imunohistochemistry shows chromogranin A is positive in carcinoid; D: The Ki-67 index in carcinoid is lower; E: The Ki-67 index in atyical carcinoid is higher; F: The morphology in carcinoid is similar as sclerosing hemangioma.

## 3 讨论

支气管肺类癌分为典型类癌和不典型类癌，两者均表现出神经内分泌分化的特征性生长方式和细胞形态学特点。根据2004年WHO病理组织学诊断标准，典型类癌和不典型类癌的区别主要在于有无坏死及核分裂像的数量。坏死的有无有时较难评价，不典型类癌的坏死常为小的点状或大的梗死样病灶。计量核分裂是一件费时且困难的工作。根据Travis的建议^[5]^，必须在肿瘤生长最活跃的区域计数核分裂，如果核分裂较少，则须扫描整张切片。固缩细胞与核分裂的区分比较困难，核分裂的细胞往往缺乏核膜，边缘较毛糙，细胞浆嗜碱性而非嗜酸性，要求确认的核分裂才能计算在内。目前认为核的不典型和多形性并不是区分两者的可靠依据。

肺的类癌还需与其它肿瘤进行鉴别诊断。类癌和微瘤型类癌的鉴别主要在大小上，后者直径 < 5 mm。在气管镜活检诊断典型类癌时，一定要结合肿瘤的大小，以防止过度诊断。肺类癌和大细胞神经内分泌癌及小细胞癌的鉴别可通过坏死、核仁和核分裂进行鉴别，后两者一般核分裂大于10个/10 HPF，如出现大面积坏死也不支持不典型类癌。三者的区别在手术和气管镜活检标本上一般不存在问题，但对某些存在明显挤压伤的小活检标本需要特别注意，因为发生机械性损伤时，细胞结构和形态看不清楚，尤其是核浆比无法判断。且当无明显的坏死及核分裂时，如果单凭酶标提示神经内分泌表达，易将肺类癌诊断为小细胞癌。这时须加做Ki-67，如果Ki-67指数偏低，诊断小细胞癌一定要慎重。类癌误诊为小细胞癌的报道亦见诸文献^[6, 7]^。有报道小细胞癌的Ki-67的指数一般 > 50%，Ki-67指数 < 20%则肯定不能诊断小细胞癌。此外，肺原发性类癌也需与转移性类癌和不典型类癌进行鉴别。除有肿瘤病史外，TTF1、CK7和CK20的组合测定亦可用于鉴别胃肠道来源且分化好的转移性神经内分泌肿瘤。既往TTF1在支气管肺类癌中的表达分歧较多，近来Rosa等^[8]^文献报道肺内的神经内分泌细胞可表达TTF1，部分肺类癌表达TTF1，非肺源的分化好的神经内分泌肿瘤往往TTF1阴性。Du等^[9]^报道TTF1只在原发性肺类癌中表达且主要表达在周围型病变中。Schmitt等^[10]^复习了604例胃肠胰肿瘤，发现仅有0.7%TTF1阳性。此外，CK7多数表达在肺肿瘤，而CK20多表达在胃肠道^[11]^，依据上述指标，基本上已可甄别类癌的原发部位。肺内发生的类癌和不典型类癌有时需与不典型硬化性血管瘤鉴别（图 1F），尤其是在术中冰冻诊断中。大体上两者境界均较清楚，切面可为灰白暗红色，质地均可为中等。显微镜下硬化性血管瘤首先要注意寻找其组织结构的复杂性，其次硬化性血管瘤的假血管瘤样区主要是不规则形状的的血池，而不是真正的扩张血管。而类癌、不典型类癌的组织结构相对单一，它的间质血管多数为血窦，或为小的薄壁血管，且小血管较少发生明显的扩张充血。硬化性血管瘤和类癌、不典型类癌的细胞均可有轻-中等度的异型，但硬化性血管瘤一般无核分裂，而类癌和不典型类癌可有核分裂（< 2/10 HPF和2-10/10 HPF）。此外，硬化性血管瘤几乎看不到坏死，类癌一般也无坏死，而不典型类癌可有点灶状坏死。免疫酶标检测可资明确鉴别两者，硬化性血管瘤间质的小圆细胞CK常常阴性，而阳性主要在乳头样区域；而类癌、不典型类癌的肿瘤细胞一般CK均阳性。硬化性血管瘤TTF1阳性；而类癌、不典型类癌TTF1阳性率偏低。更重要的是，由于类癌、不典型类癌因为是神经内分泌肿瘤，所以一般CGA、SYN、CD56阳性，须注意的是硬化性血管瘤CD56有时可阳性。类癌出现假腺样排列时，可被误诊为腺癌、粘液表皮样癌和腺样囊性癌。但腺癌一般异型性更明显，且神经内分泌标记一般阴性。此外，类癌出现器官样结构时，可与罕见的副节瘤混淆，但CK在副节瘤中一般阴性，而类癌中经常阳性。与罕见的血管球瘤鉴别时可用SMA。类癌有时细胞浆染色较淡或空，这时需与肺的透明细胞肿瘤（糖瘤）和肾透明细胞癌转移进行鉴别诊断。单凭HE形态在冰冻中几乎无法鉴别，免疫酶标是最常用的手段。糖瘤CK阴性，VIM阳性，最重要的是HMB45阳性，S100阴性。肾透明细胞癌往往有肿瘤病史，而且CK和VIM阳性，CK7阴性，神经内分泌指标阴性；而类癌、不典型类癌一般CK、CGA、SYN和CD56阳性。梭形细胞类癌需要和各种间叶源性肿瘤鉴别，可通过识别颗粒状染色质和器官样的排列以及免疫酶标鉴别。本组32例肺类癌患者中，典型类癌18例；不典型类癌14例，两者的构成和文献报道略有不同，这可能与不同医院的病例选择差异有关。Fink等^[12]^报道的168例以色列肺类癌患者中，128（76.2%）例为典型类癌；Davini等^[13]^报道89例意大利肺类癌患者63（70.8%）例为典型类癌。本组男性患者22例，女性10例，男女性别比为2.2:1。且典型类癌和不典型类癌中均为男性发病较高。Yao等^[14]^报道1977年-2004年间的35, 618例人各脏器类癌中，肺类癌的女性发病率比男性略高。Davinci等^[13]^报道男女比例基本相同。Fink等^[12]^报道男女之比为1:1.6。Han等^[15]^总结了2003年-2010年12例肺和7例胸腺不典型类癌患者男女之比为12:7。本组患者发病年龄为9岁-76岁，平均年龄43.7岁。其中典型类癌平均年龄42.9岁，不典型类癌平均年龄44.8岁，两者无统计学差异（*P*=0.729），明显低于Yao等^[14]^报道的肺类癌的平均发病年龄（62岁），Asamura等^[16]^报道中位年龄分别为52岁和63岁。究其原因可能跟种族有关，也可能跟诊断年代的早晚有关。本组近1/2的类癌患者为体检时偶然发现的，最常见的是咳嗽和咯血等与支气管阻塞有关的症状，与多数文献^[17]^一致。本组肿瘤最大径为1 cm-6 cm，平均为3.1 cm，其中典型类癌平均大小2.9 cm，不典型类癌平均大小3.3 cm，两者无统计学差异（*P*=0.366）。Asamura等^[16]^报道的类癌的平均尺寸是2.6 cm，与本组结果类似。

类癌生长缓慢，症状可多年迁延，取决于肿瘤的部位和大小。不全阻塞出现咳嗽，喘息或反复肺部感染；完全阻塞则有胸痛，发热及呼吸困难。反复咯血亦为常见。约2%的患者伴发类癌综合征（carcinoid syndrome），且多为肺部较大肿块或肝转移者出现。其它内分泌症状尚有Cushing’s syndrome、色素沉着症（MSH分泌）、低血糖症等。在诊断方面，一般胸片可无异常或仅见间接征象。CT扫描见典型类癌多为中央型腔内肿块，有气道狭窄，破坏或阻塞，约30%可见弥散点状钙化，注射增强剂可加强显像。而不典型类癌多为周围型病变，一般毗邻支气管旁，增强剂加强显像不明显且轮廓不规则。支气管镜可窥及75%的病灶，但活检易出血。类癌的自然病程较长，典型类癌术后5年生存率达95%-100%。不典型类癌有淋巴结转移者5年生存率少于60%，但切除淋巴结并术后放疗者，5年生存率可达73%。Davini^[13]^报道的89例类癌患者10年和15年的总生存率为92%和82%。Fink^[12]^报道142例类癌、不典型类癌的5年和10年生存率分别为89%、75%和82%、56%。Yao^[14]^报告30年间肺类癌的总体平均生存时间是193个月。与之相比，本组患者预后较好，15例类癌患者的5年无进展生存率100%，12例不典型类癌也高达92.9%，但随访时间相对较短。ESMO^[18]^建议肺的类癌至少应随访15年。基于肺类癌手术预后相当满意，在临床决策上应持积极态度。典型类癌仅10%-15%有淋巴结转移，非典型类癌约占类癌的10%，具侵袭性，50%-70%患者有淋巴结或远隔转移，两者对化疗均不敏感。由于大多数类癌仅有局部侵袭性，故一般主张尽可能完整切除原发病灶，同时尽量保留健肺。方法包括：①内窥镜下切除术：适宜于腔内较小肿块或不能耐受手术者的减状治疗，切除不彻底易复发；②手术切除：根据病变部位和范围，采用全肺，肺叶（含袖式），肺段，楔形切除等手术方式。术中应活检可疑淋巴结，如有转移，须扩大手术，包括纵隔淋巴结系统清扫。支气管切开取瘤或支气管局部切除偶用于息肉样病变；③放疗：能手术者不予考虑，但对无法手术的患者或不典型类癌术后可以应用；④化疗：不典型类癌有纵隔淋巴结转移，手术的同时可化疗，通常并纵隔放疗。

## 4 结论

肺类癌是低到中度恶性的神经内分泌肿瘤，典型的神经内分泌肿瘤的病理形态学特点伴CK阳性，CGA、SYN、CD56不同程度的表达以及Ki-67指数的高低是鉴别诊断的要点。鉴于较好的疗效和预后，积极争取手术是适宜的临床决策。

## References

[1] Chen LC, Travis WD, Krug LM (2006). Pulmonary neuroendocrine tumors: what (little) do we know?. J Natl Compr Canc Netw.

[2] Yao JC, Hassan M, Phan A (2008). One hundred years after carcinoid: epidemiology of and prognostic factors for neuroendocrine tumors in 35, 825 cases in the United States. J Clin Oncol.

[b3] 3Travis WD, Brambilla E, Müller-Hermelink HK, *et al*. Pathology and genetics of tumours of the lung, pleura, thymus and heart. In World Health Organization classification of tumours. IARC Press; 2004.

[4] Detterbeck FC, Boffa DJ, Tanoue LT (2009). The new lung cancer staging system. Chest.

[5] Travis WD, Rush W, Flieder DB (1998). Survival analysis of 200 pulmonary neuroendocrine tumors with clarification of criteria for atypical carcinoid and its separation from typical carcinoid. Am J Surg Pathol.

[6] Pelosi G, Rodriguez J, Viale G (2005). Typical and atypical pulmonary carcinoid tumor overdiagnosed as small-cell carcinoma on biopsy specimens: a major pitfall in the management of lung cancer patients. Am J Surg Pathol.

[7] Iyoda A, Hiroshima K, Moriya Y (2004). Pulmonary large cell neuroendocrine carcinoma demonstrates high proliferative activity. Ann Thorac Surg.

[8] Rosa SL, Chiaravalli AM, Placidi C (2010). TTF1 expression in normal lung neuroendocrine cells and related tumors: immunohistochemical study comparing two different monoclonal antibodies. Virchows Arch.

[9] Du EZ, Goldstraw P, Zacharias J (2004). TTF-1 expression is specific for lung primary in typical and atypical carcinoids: TTF-1-positive carcinoids are predominantly in peripheral location. Hum Pathol.

[10] Schmitt AM, Riniker F, Anlauf M (2008). Islet 1 (Isl1) expression is a reliable marker for pancreatic endocrine tumors and their metastases. Am J Surg Pathol.

[11] Cai YC, Banner B, Glickman J (2001). Cytokeratin 7 and 20 and thyroid transcription factor 1 can help distinguish pulmonary from gastrointestinal carcinoid and pancreatic endocrine tumors. Hum Pathol.

[12] Fink G, Krelbaum T, Yellin A (2001). Pulmonary carcinoid: presentation, diagnosis, and outcome in 142 cases in Israel and review of 640 cases from the literature. Chest.

[13] Davini F, Gonfiotti A, Comin C (2009). Typical and atypical carcinoid tumours: 20-year experience with 89 patients. J Cardiovasc Surg (Torino).

[14] Yao JC, Hassan M, Phan A (2008). One hundred years after "carcinoid": epidemiology of and prognostic factors for neuroendocrine tumors in 35, 825 cases in the United States. J Clin Oncol.

[15] Han B, Sun JM, Ahn J S (2013). Clinical outcomes of atypical carcinoid tumors of the lung and thymus: 7-year experience of a rare malignancy at single institute. Med Oncol.

[16] Asamura H, Kameya T, Matsuno Y (2006). Neuroendocrine neoplasms of the lung: a prognostic spectrum. J Clin Oncol.

[17] Travis WD (2009). Lung tumours with neuroendocrine differentiation. Eur J Cancer.

[18] Öberg K, Hellman P, Ferolla P (2012). Neuroendocrine bronchial and thymic tumors: ESMO Clinical Practice Guidelines for diagnosis, treatment and follow-up. Ann Oncol.

